# Ultrasound Evaluation of Upper Extremity Deformity

**DOI:** 10.5811/westjem.2015.11.29002

**Published:** 2016-01-12

**Authors:** Adam Janicki, Otto Liebmann

**Affiliations:** Alpert Medical School of Brown University, Department of Emergency Medicine, Providence, Rhode Island

## CASE

**Figure 1:**
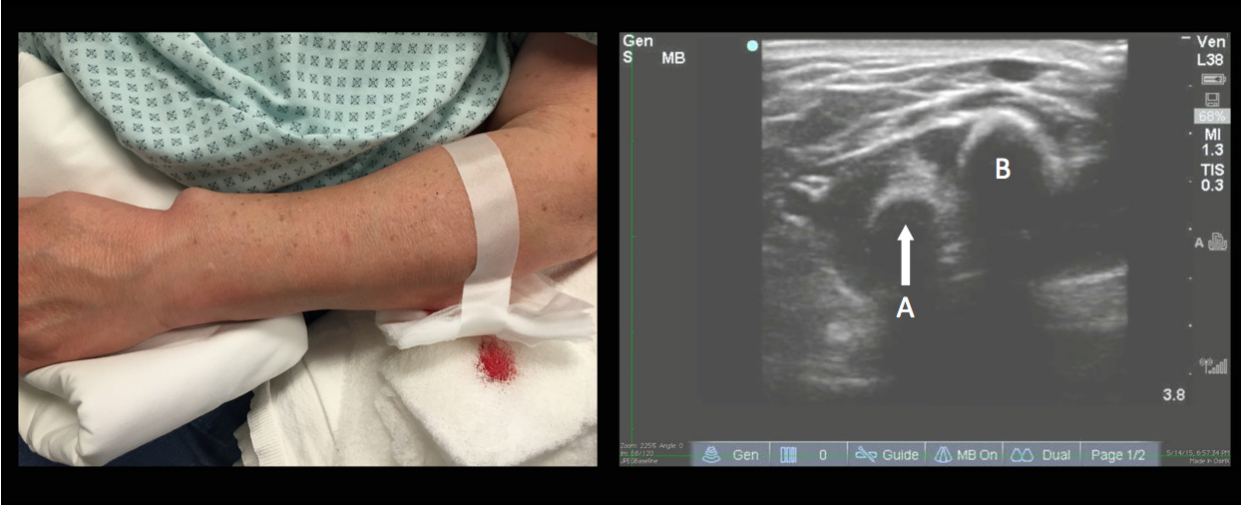
Open left forearm deformity with sagittal ultrasound image of the left forearm demonstrating a foreign body (A) radial to the ulna (B).

A 64-year-old woman presented to the emergency department after falling when she tripped on a rock while doing yard work. Physical examination revealed an open deformity of the left forearm (Figure 1). Radial pulse was palpable, sensation was intact, and she had normal range of motion of the fingers. While awaiting radiographs, bedside ultrasound was performed (Video).

**Figure 2:**
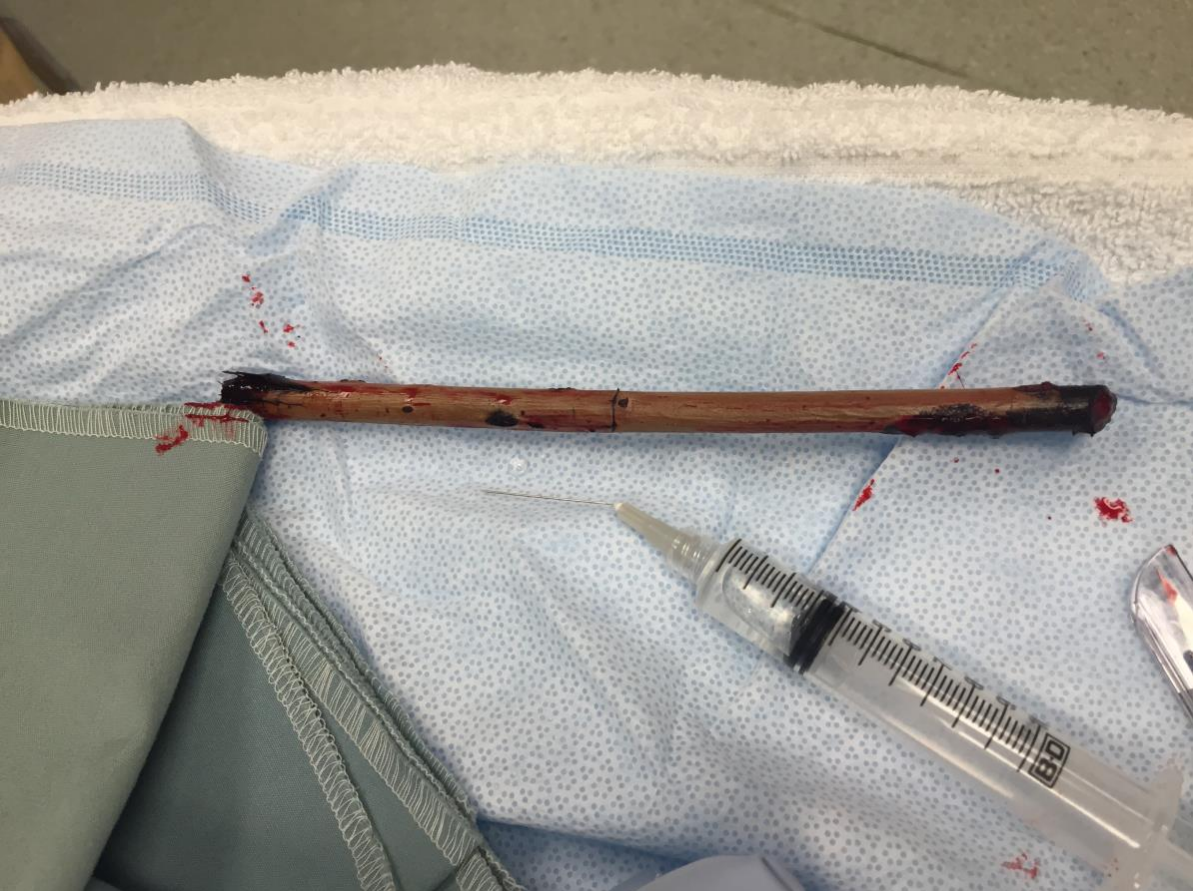
Wooden foreign body removed from the left forearm.

Ultrasound revealed intact radius and ulna and a large linear foreign body. The wooden foreign body was removed at the bedside (Figure 2) and patient was admitted for observation and intravenous antibiotics.

## DISCUSSION

Wounds containing foreign bodies are at increased risk of delayed healing and infection. Wood, in particular, is extremely inflammatory and should be removed.^1^ Although radiopaque materials (metal, glass) are often visualized on plain radiography, radiolucent objects such as wood are often not.^2^ Sensitivity of plain films for the detection of wooden foreign bodies is estimated as low as 15%.^3^

The accuracy and availability of sonography make it an excellent modality for evaluation of foreign bodies.^4^ Sensitivity of ultrasound for the detection of foreign bodies is estimated between 50% to 100%, increasing when clinical information is available.^5^,^6^ The linear, high-frequency transducer is best for examining the superficial soft tissues. Most foreign bodies are hyperechoic with a surrounding hypoechoic area corresponding to granulation tissue, edema, or hemorrhage.^7^ Foreign body size estimation is often dependent on its orientation in relation to the ultrasound beam and can be affected by local tissue reaction.^5^ Multiple tissue planes may disguise a foreign body or give the appearance of one when none is there. Air can limit the penetration of ultrasound waves or itself masquerade as a foreign body.^5^

Literature supports ultrasound’s effectiveness in evaluating for fracture, and our case demonstrates the potential of emergency physician-performed ultrasound in the evaluation of all injured extremities.^8^,^9^

## Figures and Tables

**Video:** Ultrasound of the patient’s left forearm performed with a linear probe in the sagittal view demonstrating an intact radius and ulna and a large linear foreign body.
